# Development of tetracycline-modified nanoparticles for bone-targeted delivery of anti-tubercular drug

**DOI:** 10.3389/fbioe.2023.1207520

**Published:** 2023-08-10

**Authors:** Qiuzhen Liang, Pengfei Zhang, Liang Zhang, Haopeng Luan, Xinxia Li, Haibin Xiang, Shuang Jing, Xinghua Song

**Affiliations:** ^1^ Sports Medicine Center, Honghui Hospital, Xi’an Jiaotong University, Xi’an, Shaanxi, China; ^2^ Department of Gastroenterology, XD Group Hospital, Xi’an, Shaanxi, China; ^3^ Department of Spine Surgery, The Sixth Affiliated Hospital of Xinjiang Medical University, Urumqi, China; ^4^ School of Pharmacy, Xinjiang Medical University, Urumqi, China; ^5^ Department of Orthopaedics, The First Affiliated Hospital of Xinjiang Medical University, Urumqi, China

**Keywords:** rifapentine, bone targeting, nanoparticles, osteoarticular tuberculosis, anti-tubercular

## Abstract

**Background:** Since the poor response to existing anti-tuberculosis drugs and low drug concentration in local bone tissues, the traditional drug therapy does not result in satisfactory treatment of osteoarticular tuberculosis. Thus, we report a rifapentine release system with imparted bone targeting potential using tetracycline (TC) -modified nanoparticles (NPs).

**Methods:** TC was conjugated to PLGA-PEG copolymer via a DCC/NHS technique. Rifapentine-loaded NPs were prepared by premix membrane emulsification technique. The resulting NPs were characterized in terms of physicochemical characterization, hemolytic study, cytotoxicity, bone mineral binding ability, *in vitro* drug release, stability test and antitubercular activity. The pharmacokinetic and biodistribution studies were also performed in mice.

**Results:** Rifapentine loaded TC-PLGA-PEG NPs were proved to be 48.8 nm in size with encapsulation efficiency and drug loading of 83.3% ± 5.5% and 8.1% ± 0.4%, respectively. The release of rifapentine from NPs could be maintained for more than 60 h. Most (68.0%) TC-PLGA-PEG NPs could bind to HAp powder *in vitro*. The cellular studies revealed that NPs were safe for intravenous administration. *In vivo* evaluations also revealed that the drug concentration of bone tissue in TC–PLGA–PEG group was significantly higher than that in other groups at all time (*p* < 0.05). Both NPs could improve pharmacokinetic parameters without evident organ toxicity. The minimal inhibitory concentration of NPs was 0.094 μg/mL, whereas this of free rifapentine was 0.25 μg/mL.

**Conclusion:** Rifapentine loaded TC-PLGA-PEG NPs could increase the amount of rifapentine in bone tissue, prolong drug release in systemic circulation, enhance anti-tuberculosis activity, and thereby reducing dose and frequency of drug therapy for osteoarticular tuberculosis.

## Introduction

Tuberculosis is an infectious bacterial disease, and remains a global health problem with rising morbidity and mortality. According to the global tuberculosis report 2022, Tuberculosis has surpassed HIV as the most lethal infectious disease in the world (World Health Organization, 2022). Osteoarticular tuberculosis accounts for 35% of extrapulmonary tuberculosis. Since the poor response to existing anti-tuberculosis drugs and low drug concentration in local bone tissues, the traditional drug therapy does not result in satisfactory treatment of osteoarticular tuberculosis (Wang B. et al., 2021). In addition, the poor permeation of anti-tuberculosis agents necessitates long term administration of high drug doses to maintain concentration in local bone tissue. Therefore, the traditional oral treatment for osteoarticular tuberculosis involves high daily doses of drugs for at least 12 months. (Li et al., 2016). Unfortunately, after a short while of starting drug therapy, most patients complain about severe side effects, and some of them drop out early therapy, resulting in a low patient compliance and even the emergency of multidrug-resistant tuberculosis.

Amongst first-line anti-tuberculosis drugs, rifampicin suffers from various drawbacks like short half-life, poor bioavailability and high hepatotoxicity, leading to subtherapeutic levels of rifampicin in blood and increased risk of multidrug-resistant tuberculosis (Toft et al., 2022; World Health Organization, 2022). In contrast, rifapentine is a rifamycin derivative with a half-life and anti-tubercular several times greater than that of rifampicin (Zumla et al., 2015). Although the composite scaffold containing rifapentine was developed in our previously reported works and implanted into bone defect, repeated administration was not possible (Wang Z. et al., 2021). Thus, to develop a delivery system that can reduce both the dose and frequency of drug while improving therapeutic effect in local bone tissue appears to be the most promising option for long-term drug treatment of osteoarticular tuberculosis.

The current strategy for enhancing the therapeutic activity of currently available drugs is to entrap drugs within a delivery system. Drug delivery systems have been introduced to increase the permeability, solubility and metabolic stability of a drug molecule. Amongst various systems, nanoparticles (NPs) offer potential advantages over free drug, including increasing therapeutic efficacy and prolonging drug release (Sukhithasri et al., 2014). Polymeric NPs have been widely used in clinical treatment because of their good biocompatibility, and their byproducts can be eliminated through normal metabolic pathways (Luque-Michel et al., 2017). Amongst all biomaterials, PLGA (poly-d,l-lactide-co-glycolide) have been approved by Food and Drug Administration (FDA) for biomedical applications due to biodegradability nature (Mir et al., 2017; Kim et al., 2019), and it may be a promising material for targeting, imaging, and therapy. In addition, PEG (poly ethylene glycol) can further provides a steric barrier to prolong NPs circulation (Xu et al., 2015).

In the present study, in order to give NPs the ability to prolong the circulation time as well as target bone tissue, a tetracycline (TC)-modified drug delivery system was developed. Molecules such as TC have been used as a group to target bone tissue because of their unique hydroxyapatite affinity (Chu et al., 2017). TC itself is also a broad spectrum antibiotic used to treat a variety of bacterial infections. To the best of our knowledge, the bone-targeted nano-formulations of anti-tuberculosis drug has not yet been reported in literature. We hope that these bone targeted NPs would increase the amount of rifapentine in bone tissue, prolong drug release, enhance anti-tuberculosis activity while diminish organ toxicity, and thereby allowing less dosage and frequency.

## Materials and methods

### Materials

All chemicals and reagents were purchased from Sigma Chemical (St. Louis, Missouri, United States) unless otherwise stated. Hydroxyapatite (HA) powder was purchased from Chemical Reagent Co., Ltd. (Shanghai, China). Rifapentine (RPT) (content: 98%) was obtained from Sichuan Med-Shine Pharmaceutical Co., Ltd. (Sichuan, China). PLGA-PEG-COOH (Mw: 12,000; lactide:glycolide = 50:50) was purchased from Jinan Daigang Biomaterial Co., Ltd. (Shandong, China). Dicyclohexylcarbodiimide (DCC) and N-Hydroxysuccinimide (NHS) were obtained from Xiya Chemical Technology Co., Ltd. (Shandong, China). TC was purchased from Shanghai Yuanye Biological Technology Co., Ltd. (Shanghai, China). 3-[4,5-dimethylthiazol-2-yl]-2,5-diphenyl tetrazolium bromide (MTT) cytotoxicity assay kit was purchased from Bejing Solarbio Science and Technology Co., Ltd. (Bejing, China). Dubelcco’s Modified Eagle’s Medium (DMEM), trypsineEDTA, fetal bovine serum (FBS) and penicillin-streptomycin solution were obtained from Thermo Fisher Scientific (Waltham, MA, United States). 1,1′-Dioctadecyl-3,3,3′,3′-tetramethylindotricarbocyanine iodide (DiR) was obtained from Abcam (Cambridge, United Kingdom). Shirasu porous glass (SPG) membranes were purchased from SPG Technology Co. Ltd. (Miyazaki, Japan).

### Synthesis of TC-PLGA-PEG conjugates

**FIGURE 1:**
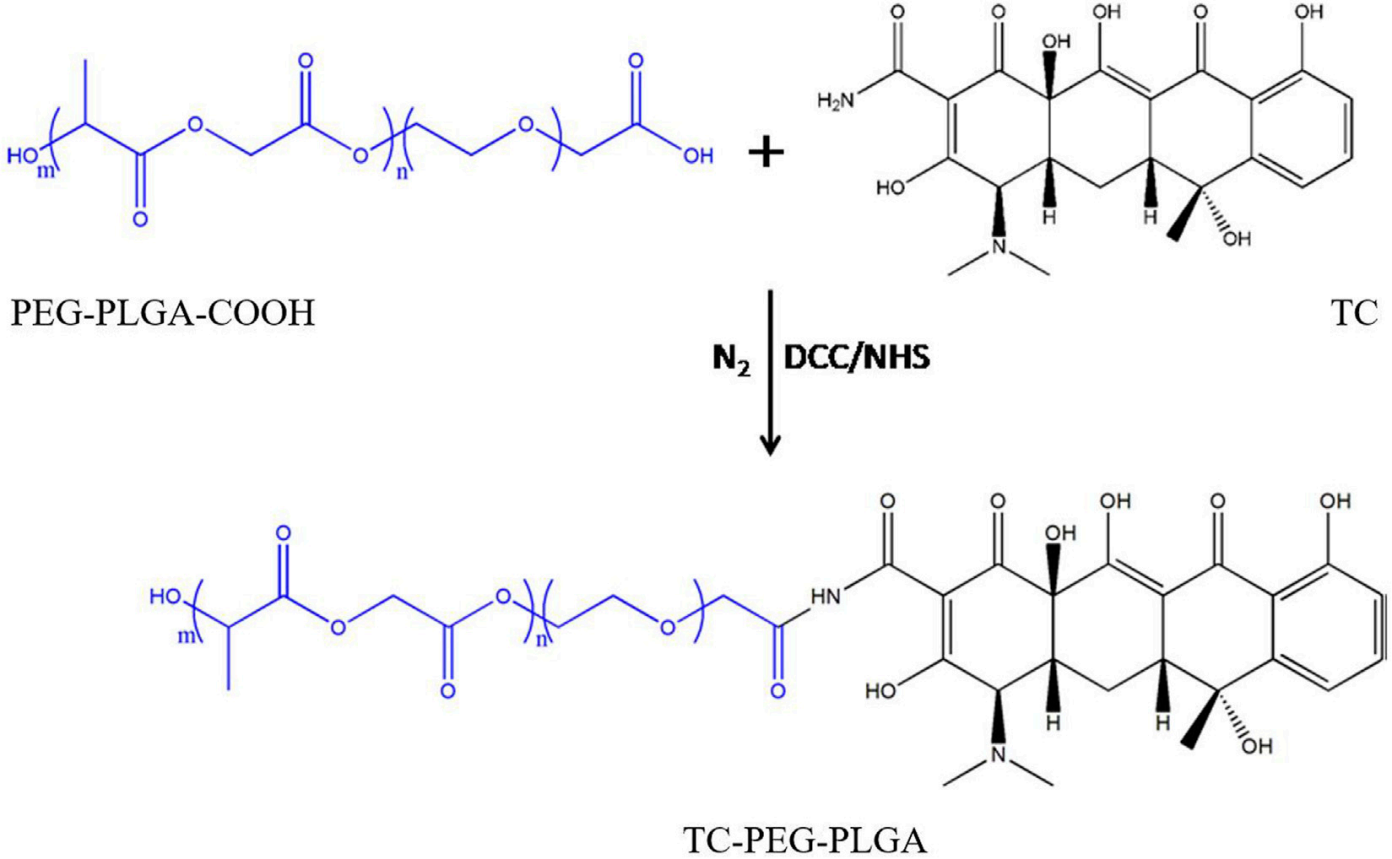
The synthesis route of TC-PLGA-PEG conjugates.

A DCC/NHS technique was used to synthesize the TC-PLGA-PEG conjugates (Figure 1). Briefly, 500 mg of PLGA-PEG-COOH, 25.8 mg of DCC, and 14.5 mg of NHS were dissolved in 30 mL anhydrous dimethyl sulfoxide (DMSO). This solution was stirred for 24 h to activate the carboxylic acid under the protection of nitrogen. The by-product was removed using a syringe filter with 0.45 μm pore size. Then, 18.5 mg of TC was added to the activated PLGA-PEG, the mixture was sequentially stirred under the protection of nitrogen for 24 h at 60°C. The resultant solution was dialyzed against deionized water using a dialysis membrane (MWCO 3.5 kDa) for another 48 h, and then centrifuged at 18,000 rpm for 15 min. The resultant solution washed three times with deionized water. The final product was lyophilized and stored.

### Characterization of TC-PLGA-PEG conjugates

To evaluate molecular state of the product, UV-2550 spectrophotometer spectra (Shimadzu, Japan) of TC, PLGA-PEG and TC-PLGA-PEG were recorded with DMSO as the solvent. Then, the product was further confirmed by Fourier transform infrared (FTIR) spectra (Shimadzu, Japan). The spectra of TC, PLGA-PEG and PLGA-PEG-TC between the wavelengths of 500 and 4,000 cm^−1^ were recorded. In addition, those conjugates were further confirmed by ^*1*^ *H* NMR spectra on Bruker spectrometer.

### Preparation of drug-loaded bone-targeted NPs

**FIGURE 2:**
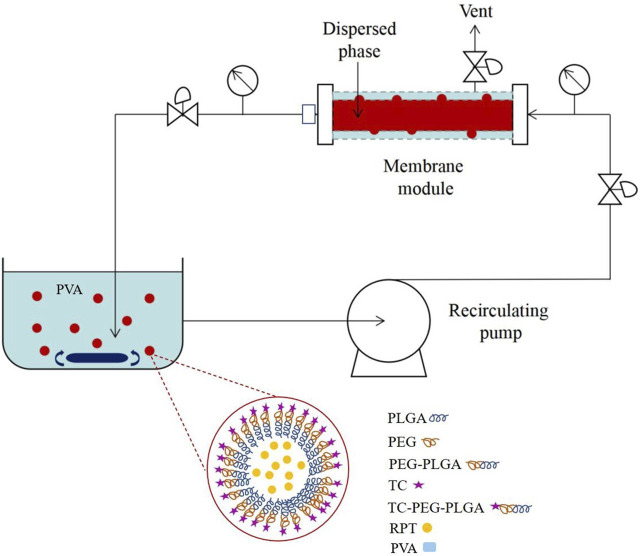
Schematic diagram of drug-loaded bone-targeted NPs.

Drug-loaded bone-targeted NPs (rifapentine/TC-PLGA-PEG NPs) were prepared by premix membrane emulsification technique (Figure 2). At first, aqueous solution was mixed with DCM containing rifapentine and TC-PLGA-PEG, and the mixture was sonicated using a probe sonicator (UP400S, Hielscher Ultrasonics, Germany) at 50% amplitude for 1 min to form primary emulsion. It was then coarsely emulsified through SPG membrane (1 μm pore size, SPG Technology Co., Ltd., Sadowaracho, Japan) under magnetic stirring for 5 min to form coarse emulsion. Secondly, this coarse emulsion was subjected to five homogenization cycles by the usage of SPG membrane (0.1 μm pore size) with a high pressure. The obtained emulsion was poured quickly into 100 mL of a 1% (w/v) PVA solution and stirred overnight to evaporate organic solvent. Afterwards, the solutions were filtered through the 0.45-μm microporous membrane. The separated NPs were collected by centrifugation and washed for three times followed by lyophilization. The non-targeted NPs (rifapentine/PLGA-PEG NPs) were obtained using the same method. To make fluorescent-labelled NPs, coumarin 6 or DIR was added to the DCM, as the only change to the procedure described above.

### Physical and chemical characterization

The zeta potential (ZP), polydispersity index (PDI) and particle size (PS) were determined using a ZetaSizer Nano-ZS (Malvern Instruments Ltd., Malvern, United Kingdom). The morphology of rifapentine/TC-PLGA-PEG NPs was confirmed using a transmission electron microscopy (JEM-1230, Jeol Ltd., Japan) after staining with sodium phosphotungstate solution (2%, w/v). The drug entrapment efficiency (EE%) and drug loading (DL%) were assayed by ultraviolet spectrophotometry method at a wavelength of 475 nm (Wu et al., 2015). To assess the stability of NPs, samples were stored in a stable chamber at 25°C for 90 days. Periodically, a sample was collected to measure PDI, EE% and particle size.

### *In vitro* drug release

*In vitro* rifapentine release study was evaluated by dialysis method. Briefly, free rifapentine, rifapentine/PLGA-PEG and rifapentine/TC-PLGA-PEG NPs were placed in a dialysis bag (MWCO 3.0 kDa), followed by incubated in PBS medium (pH 7.4). The PBS medium was maintained at 37°C and stirred at 100 rpm. At pre-determined intervals, samples were withdrawn and replaced by the same volume of fresh medium. The content of rifapentine was measured using ultraviolet spectrophotometry method as described above.

### HAp affinity assay

To evaluate the bone mineral binding ability, HAp powder was used as previously reported (Xie et al., 2018). The coumarin 6-labelled rifapentine/PLGA-PEG and rifapentine/TC–PLGA–PEG NPs (5 mL, 0.1 wt%) were detected via fluorospectrophotometry before reaction. Then, the solutions were separately added into PBS containing HAp (10 mg/mL) and shaken in the dark for 2 h. After filtrated with 0.22 μm microporous membrane, the intensity of the supernatant was detected. The binding ratio (%) in relation to initial intensity was calculated using the following equation: $Binding\;ratio\;\left(\%\right)=\left[\left(I_{intensity\;before\;Hap\;adsorption}-I_{intensity\;after\;Hap\;adsorption}\right)/I_{intensity\;before\;Hap\;adsorption}\right]\times 100$

### Cytotoxicity study

Bone marrow mesenchyml stem cell (BMSCs) were obtained from 12-week-old New Zealand white rabbits according to our previous report (Wang et al., 2015). The cytotoxicity of NPs was evaluated by MTT assay. Rifapentine solution, rifapentine/PLGA-PEG NPs and rifapentine/TC-PLGA-PEG NPs were incubated with BMSCs at 37°C for 24 h at final RPT concentrations of 0, 5, 25 and 50 μg/mL. Afterwards, 15 μL of MTT was added to each well and incubated further for another 4 h. After dissolving and shaking, the absorbance of formazan at 570 nm was measured with a microplate reader. Furthermore, different concentrations of blank NPs (ranging from 0 μg/mL to 1,200 μg/mL) were also incubated with BMSCs. The cell viability (%) in relation to negative control group was calculated using the equation:

$$viability\;\left(\%\right)=\frac{\left[A\right]\;cells\;incubated\;with\;formulation}{\left[A\right]\;cells\;incubated\;only\;with\;culture\;medium}\times 100$$

### Haemolytic study

Haemolytic toxicity was conducted to evaluate the compatibility of rifapentine loaded NPs with erythrocytes. In this study, the blood was collected from 12-week-old New Zealand white rabbits. Briefly, the erythrocytes were separated and resuspended in 0.9% w/v normal saline. Various concentrations (0.5–3.5 mg/mL) of NPs were mixed with normal saline for interaction with erythrocytes suspension. Then, a UV–visible spectrophotometer at 545 nm was used for absorbance measurements. The haemolytic rate of the sample was calculated by using the equation: $Haemolytic\;rate\;\left(\%\right)=\left[\left(A_{absorbance\;of\;sample}-A_{absorbance\;of\;saline}\right)/\;\left(A_{absorbance\;of\;water}-A_{absorbance\;of\;saline}\right)\right]\times 100$

### Anti-tuberculosis studies

The antimycobacterial activity of NPs against *M. tuberculosis* was evaluated by microplate Alamar blue assay following a previously reported protocol (Que et al., 2022). Firstly, 200 μL of sterile deionized water was added in outer peripheral wells. Secondly, 100 μL of 7H9GC-OADC broth and 100 μL of rifapentine was added separately. Thirdly, serial dilutions (1:2) were performed in columns 3 to 10. The final drug concentration range of free rifapentine was 1.0 μg/mL to 0.004 μg/mL, and the final drug concentration range of rifapentine loaded NPs was 0.75 μg/mL to 0.003 μg/mL. Finally, *M. tuberculosis* inocula were added in columns 2 to 11. Column 11 was considered as a positive control without drug. After 5 days of incubation, 50 μL of Alamar Blue reagent and 10% Tween 80 was added and re-incubated for another 24 h. The results were analysed by means of colour changing in each well. The concentration corresponding to no observable bacterial growth is the minimum inhibitory concentration of formulation.

### Pharmacokinetics study

Kunming male mice (body weight 27 ± 2 g) were obtained from the Animal Center of Xinjiang Medical University. Animal experiments and welfare were complied with the Guide for the Care and Use of Laboratory Animals published by the National Institutes of Health (No. 8023). The experiments were approved by the Ethics Committee of The Xinjiang Medical University (No 20180223–13). The mice were divided into three groups: rifapentine, rifapentine/PLGA-PEG NPs and rifapentine/TC-PLGA-PEG NPs. The mice were given a single dose of 10 mg/kg (rifapentine) intravenously. Then, blood samples were taken from the retro-orbital plexus of each animal per time point. Acetonitrile was used to precipitate the separated plasma. Then, the supernatants were removed after vortex and centrifugation. The final sample was injected on the HPLC column to estimate the amount of rifapentine. The HPLC analysis was performed using a C18 column (250 mm × 4.6 mm, 5 µm particle size; Waters, United States) at 25°C. The mobile phase consisted of methanol-0.0367 mol/L KH_2_PO_4_, flow rate of 1 mL/min, and detection at a wavelength of 254 nm. The maximum concentration (*C*_*max*_), the time to reach maximum plasma concentration (*T*_*max*_), the peak concentration (*C*_*max*_), the half-life (*t*_*1/2*_), the mean residence time (MRT), and the area under the curve (AUC_0–∞_) were evaluated.

### Tissue distribution study

For a single-dose rifapentine disposition study, the mice were given a single dose of 10 mg/kg rifapentine intravenously in each group. The mice were divided into four groups: control group (normal saline), rifapentine/PLGA-PEG NPs and rifapentine/TC-PLGA-PEG NPs. The animals were sacrificed at a specified time interval. After the plasma was obtained, their tissues (liver, kidney and bones) were excised and washed quickly with normal saline, then stored at −20°C. The drug concentration was estimated in 20% (w/v) tissue homogenates. The content of rifapentine was measured using HPLC method as described above. The results were expressed as μg/g (microgram per Gram of tissue).

To further confirm the bone-targeting efficacy of rifapentine/TC-PLGA-PEG NPs, Dir-loaded NPs were injected into the Kunming mice through the tail vein after anesthetization. The Kunming mice were randomly divided into two groups of DIR loaded rifapentine/TC–PLGA–PEG NPs, and DIR loaded rifapentine/PLGA–PEG NPs. Animals in each group were observed 48 h post injection using IVIS^®^ Spectrum *In vivo* Imaging System.

To observe the side effects of NPs on mouse organs, the animals were sacrificed at the fourth day after the injection. The heart, kidney, liver, lung and spleen were harvested, then fixed in 10% neutral formalin for 72 h. The samples were paraffin embedded for haematoxylin and eosin (H&E) staining. In addition, the blood samples were also obtained for blood biochemistry testing before the mice were euthanized.

### Statistical analysis

Statistical analysis was performed using SPSS version 17.0. Results are expressed as mean ± SD, and differences between continuous variables were assessed via One-way ANOVA with Tukey’s test. A statistically significant difference was defined as *p* < 0.05.

## Results

### Physical and chemical characterization

**FIGURE 3:**
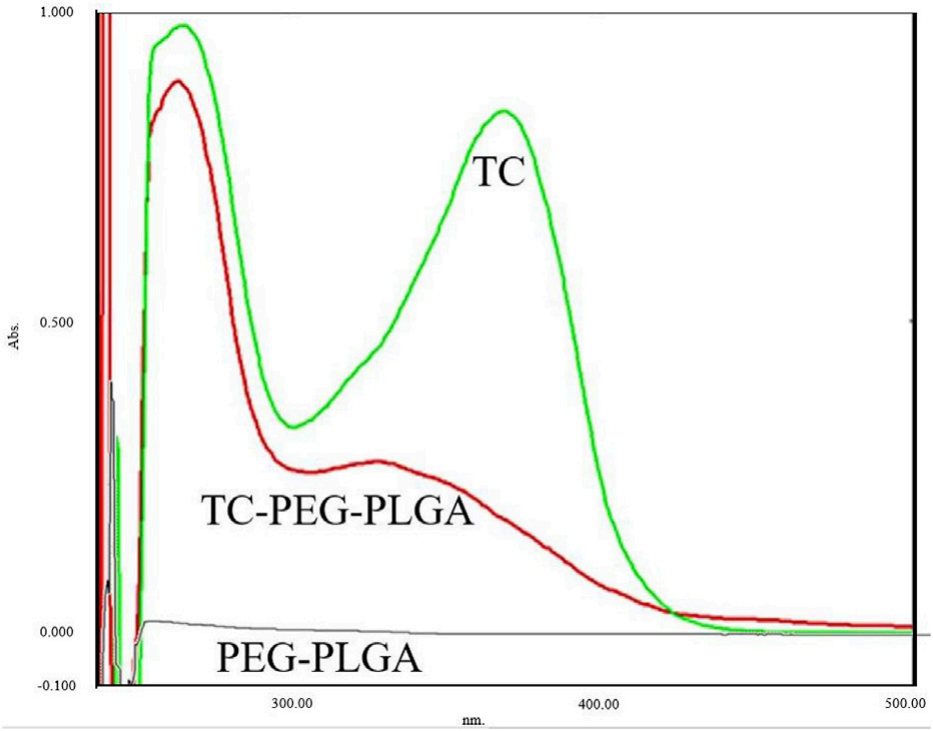
The ultraviolet spectra of TC, PLGA–PEG and TC–PLGA–PEG.

The UV spectra of TC, PLGA–PEG and TC–PLGA–PEG were shown in Figure 3. The spectrum of TC showed two characteristic absorption peaks at 266 nm and 367 nm. In contrast, no obvious UV absorption peak was observed at 200 nm–800 nm before the reaction, indicating that the mixed solution of PLGA–PEG copolymer had no obvious UV absorption peak. Compared with the spectrum of TC before the reaction, spectrum of TC-PLGA-PEG showed no significant UV absorption peak near 367 nm instead of a weak absorption peak at 328.5 nm, and the peak at 266 nm still existed. The results showed that the synthesis of TC makes PLGA–PEG solution without characteristic peaks appear obvious characteristic peaks, and these characteristic peaks are similar to the characteristic peaks of TC.

**FIGURE 4:**
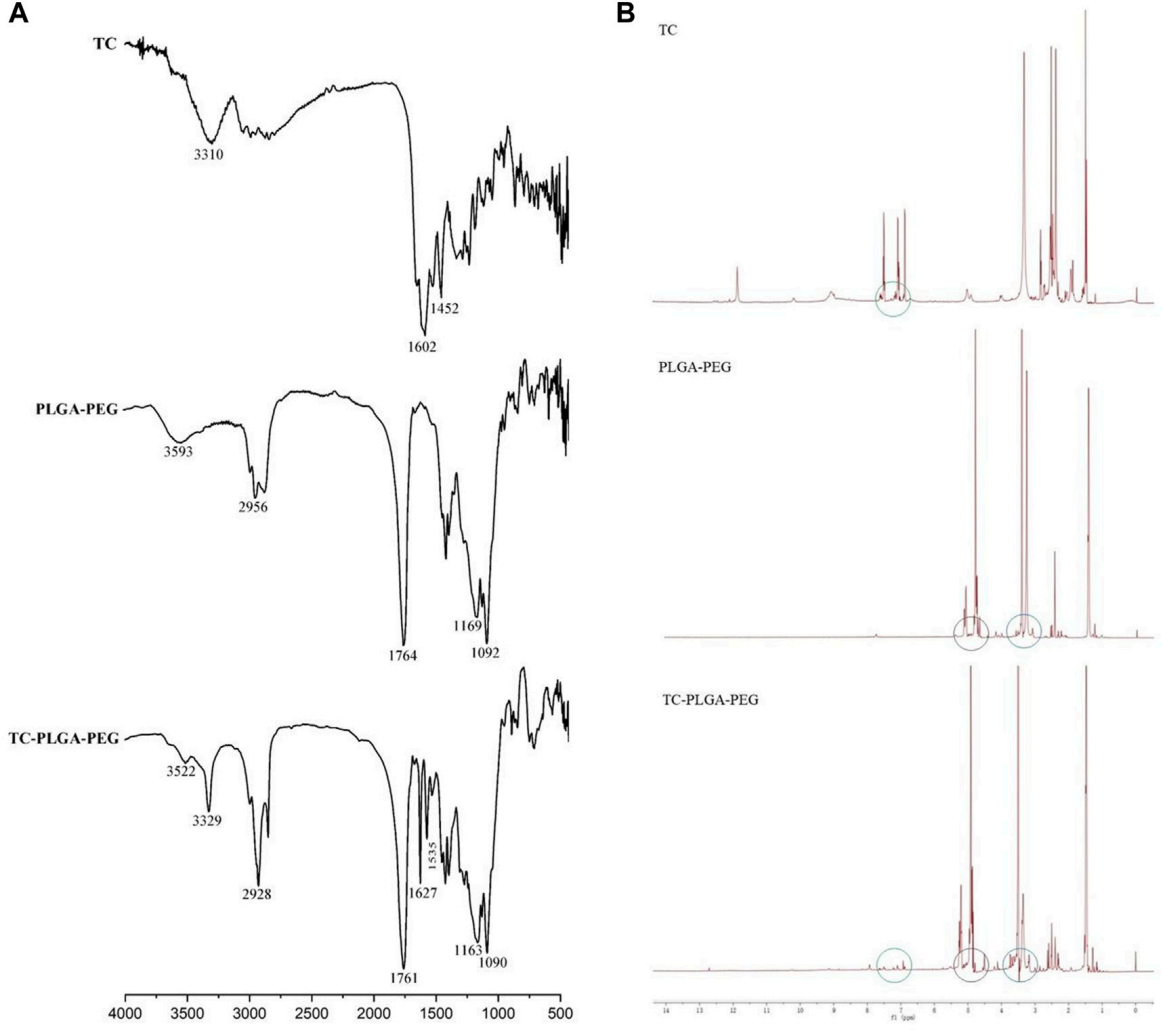
The infrared spectra **(A)** and ^*1*^ *H* NMR spectrum **(B)** of TC, PLGA–PEG and TC–PLGA–PEG.

The infrared spectra of TC, PLGA–PEG and TC–PLGA–PEG were shown in Figure 4A. In the spectrum of PLGA–PEG, the absorption band at 3,593 cm^−1^ was assigned to terminal hydroxy groups in the copolymer, and the band at 2,956 cm^−1^ was C-H stretch. A strong band at 1764 cm^−1^ was attributed to C=O stretch, and the absorption at 1,169–1,092 cm^−1^ was assigned to C-O and C-O-C groups, which are consistent with the data in the previous reports. (Ge et al., 2018). The spectrum of TC showed two particular bands at 3,310 cm^−1^ and 1,650–1,450 cm^−1^, corresponding to the N-H stretch and benzene ring, respectively. While all above mentioned peaks of PLGA–PEG and TC were presented in the spectrum of TC–PLGA–PEG conjugates as well, there were some minor changes and shifts by which it was possible to conclude the successful incorporation with each other. In the spectrum of TC–PLGA–PEG, the absorption band around 3,330 cm^−1^ was assigned to the mixed band of OH in the copolymer and NH in amido bond; The absorption band around 1760 cm^−1^ was assigned to C=O stretch in the copolymer; The absorption band around 1,630 cm^−1^ was assigned to C=O in amido bond; The absorption band around 1,530 cm^−1^ was assigned to C-N-H in amido bond; The absorption band around 1,300 cm^−1^ was mainly assigned to the mixed band of C-N and N-H in amido bond.

The ^*1*^ *H* NMR spectrum of TC, PLGA–PEG and TC–PLGA–PEG were shown in Figure 4B. The peaks at 7.0–8.0 ppm belonged to the hydrogens of the benzene ring of TC, peaks at 4.0–6.0 ppm belonged to the methylene and methylidyne hydrogens of PLGA, and peaks at 3.6 ppm belonged to the–CH2CH2O–units of PEG, respectively. These peaks are consistent with the previous reports (Wang et al., 2015; Xie et al., 2018). Based on ^*1*^ *H* NMR, UV and infrared spectrum, it was evidence that the TC-PEG-PLGA conjugates were synthesized successfully.

**FIGURE 5:**
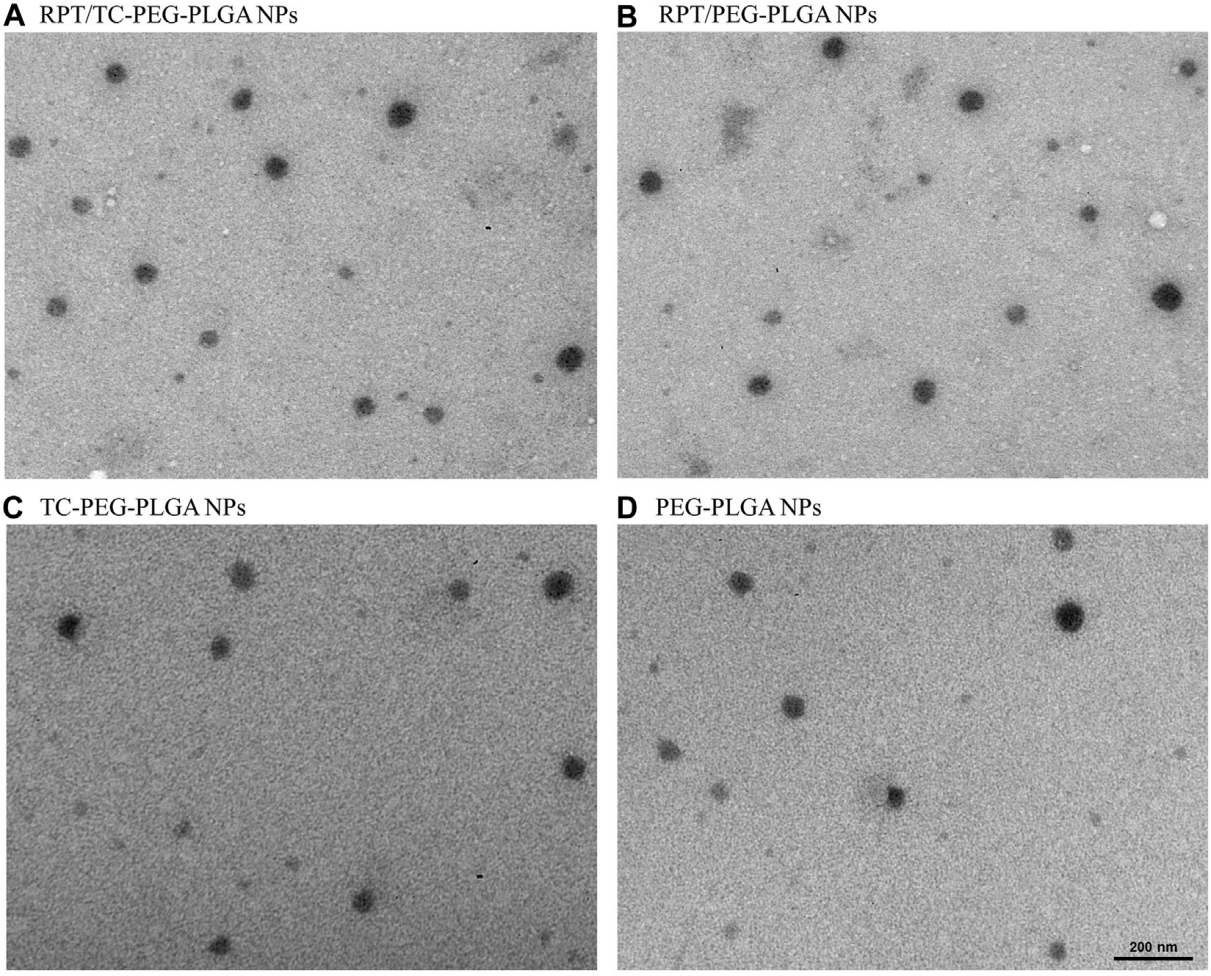
Transmission electron microscopy images of NPs **(A)** RPT/TC-PEG-PLGA NPs. **(B)** RPT/PEG-PLGA NPs. **(C)** TC-PEG-PLGA NPs. **(D)** PEG-PLGA NPs.

The particle size of RPT/TC–PLGA–PEG NPs appears to be slightly larger than RPT/PLGA–PEG NPs, but the difference is not significant. Drug encapsulating efficiency of both RPT–loaded NPs was greater than 80% with high DL%. With regard to applying single emulsifier, the NPs prepared by solvent evaporation showed noticeably sustained release behavior when 1% PVA were used, and the concentration of PVA produced NPs with high EE% (>80%). The PDI of both NPs was found to be around 0.20, suggesting that the NPs had good homogenetity (Table 1). Transmission electron microscope showed that rifapentine loaded TC-PLGA-PEG NPs, rifapentine loaded PLGA-PEG NPs, TC-PLGA-PEG NPs and PLGA-PEG NPs were dispersed as individual particles with a spherical shape (Figure 5). There were no significant changes on the surface or interior of NPs, which is consistent with the previous findings reported in the literature (Wang et al., 2015; Que et al., 2022). In addition, there were no significant differences in stability studies, indicating that both NPs were stable for at least 3 months (Table 2).

**TABLE 1:** Physicochemical characterization of rifapentine loaded NPs.

Sample	Particle size (nm)	PDI	ZP (mV)	EE (%)	DL (%)
rifapentine/PLGA-PEG NPs	47.2 ± 7.3	0.19 ± 0.07	−17.5 ± 2.3	81.0 ± 6.2	7.9 ± 0.6
rifapentine/TC-PLGA-PEG NPs	48.8 ± 5.8	0.21 ± 0.04	−16.8 ± 1.9	83.3 ± 5.5	8.1 ± 0.4

Abbreviations: TC, tetracycline; PLGA, poly-d,l-lactide-co-glycolide; PEG, poly ethylene glycol; NPs, nanoparticles; PDI, polydispersity index; ZP, zeta potential; EE, encapsulation efficiency; DL, drug loading.

**TABLE 2:** Stability evaluation.

Sample	Time	Particle size (nm)	PDI	EE (%)
rifapentine/PLGA-PEG NPs	Initial	47.2 ± 7	0.19 ± 0.07	81.0 ± 6.2
	1 month	48.1 ± 8	0.19 ± 0.06	82.4 ± 7.3
	3 months	49.4 ± 11	0.20 ± 0.07	79.1 ± 7.6
Rifapentine/TC-PLGA-PEG NPs	Initial	48.8 ± 5.8	0.21 ± 0.04	83.3 ± 5.5
	1 month	50.0 ± 8.5	0.20 ± 0.06	81.7 ± 7.4
	3 months	49.5 ± 8.2	0.21 ± 0.05	80.1 ± 8.3

Abbreviations: TC, tetracycline; PLGA, poly-d,l-lactide-co-glycolide; PEG, poly ethylene glycol; NPs, nanoparticles; PDI, polydispersity index; EE, encapsulation efficiency.

### Drug release study

**FIGURE 6:**
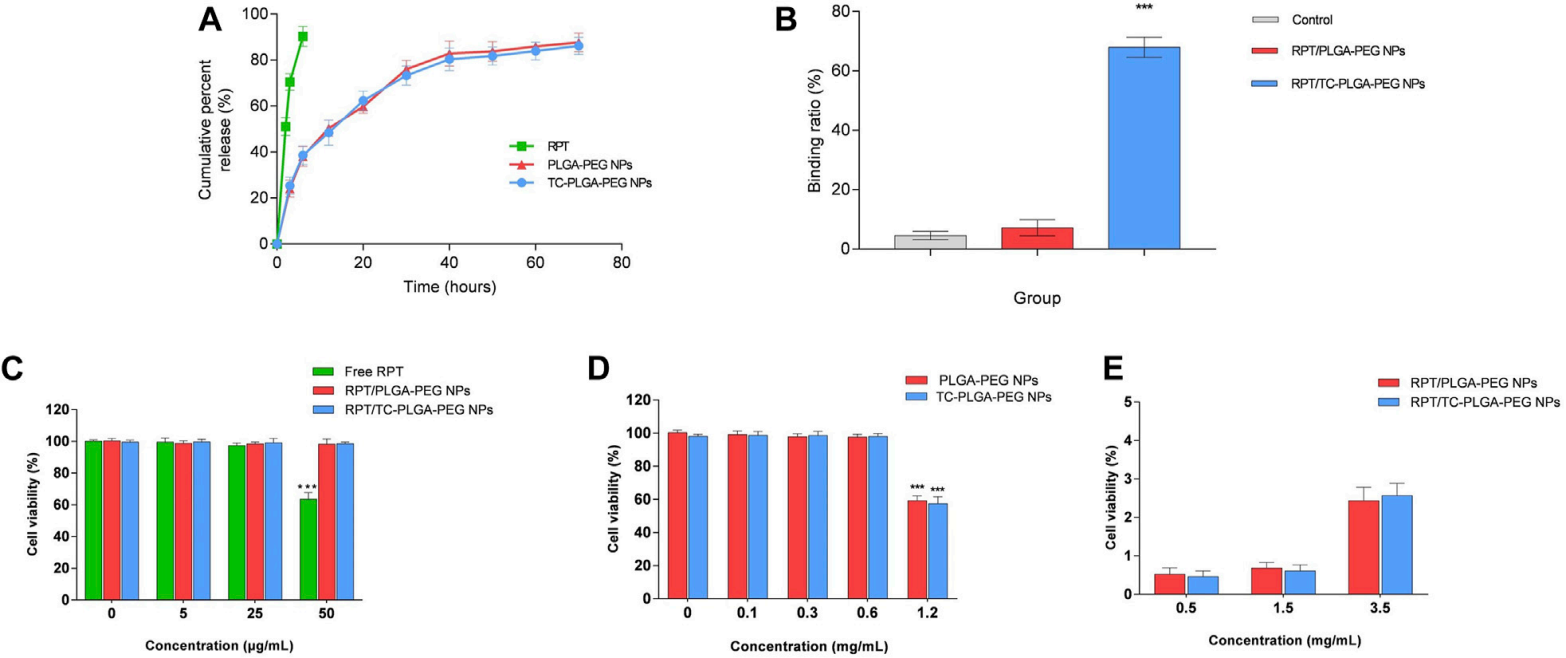
**(A)** *In vitro* cumulative release curves of free RPT, RPT/PLGA–PEG NPs and RPT/TC–PLGA–PEG NPs. **(B)** Bone mineral binding ability. **(C)** Cytotoxicity studies of drug loaded NPs on rabbit bone marrow mesenchyml stem cell. Note: ***p < 0.001 compared with the negative control. **(D)** Cytotoxicity studies of blank NPs on rabbit bone marrow mesenchyml stem cell. Note: ***p < 0.001 compared with the negative control. **(E)** Haemolytic toxicity of various concentrations of NPs.

The release mode of rifapentine from TC–PLGA–PEG and PLGA–PEG NPs was shown in Figure 6A. The release pattern was characterized by an initial burst and subsequent controlled release. By contrast, free rifapentine was released in a burst manner within 2 h, and approximately 90% in 6 h. The release kinetics of both NPs were evaluated using the zero-order equation, first-order equation, and Higuchi equation models. In the TC-PEG-PLGA group, the R square of the zero-order equation, first-order equation and Higuchi equation was 0.82084, 0.95765 and 0.93803, respectively. Similarly, in the PEG-PLGA group, the R square of the zero-order equation, first-order equation and Higuchi equation was 0.81452, 0.96099 and 0.93233, respectively. Therefore, it can be concluded that the release kinetics of both NPs follow the first-order equation model.

### HAp affinity

Figure 6B showed the binding ratio of rifapentine/PLGA-PEG and rifapentine/TC-PLGA-PEG NPs. Most (68.0%) of the rifapentine/TC-PLGA-PEG NPs bound to the HAp powder, whereas only small amounts of coumarin 6 (4.6%) and rifapentine/PLGA–PEG NPs (7.2%) were non-specifically adsorbed.

### Toxicity study

Cytotoxicity was evaluated on BMSCs. The results exhibited in Figure 6C showed that both NPs were nontoxic for BMSCs. Both drug-loaded NPs did not present a significant difference (*p* > 0.05) compared with the negative control. However, free rifapentine presented a cell viability of less than 70% at concentrations of 50 μg/mL, demonstrating that the nano-formulation may reduce the toxicity of rifapentine. To further evaluate the effect of blank NPs on cell viability, the cells were exposed to NPs at different concentrations. The results revealed that cell viability was still above 90% even at a concentration of 0.6 mg/mL. However, it must be emphasized that high concentration (1.2 mg/mL) of NPs can still affect cell activity, but in practical applications, the dose is much smaller than this concentration (Figure 6D). Furthermore, both NPs were found to possess negligible haemolysis of erythrocytes even at the highest concentrations (Figure 6E). Therefore, rifapentine loaded NPs were potentially feasible for intravenously administration.

### *In vitro* anti-tubercular studies

**FIGURE 7:**
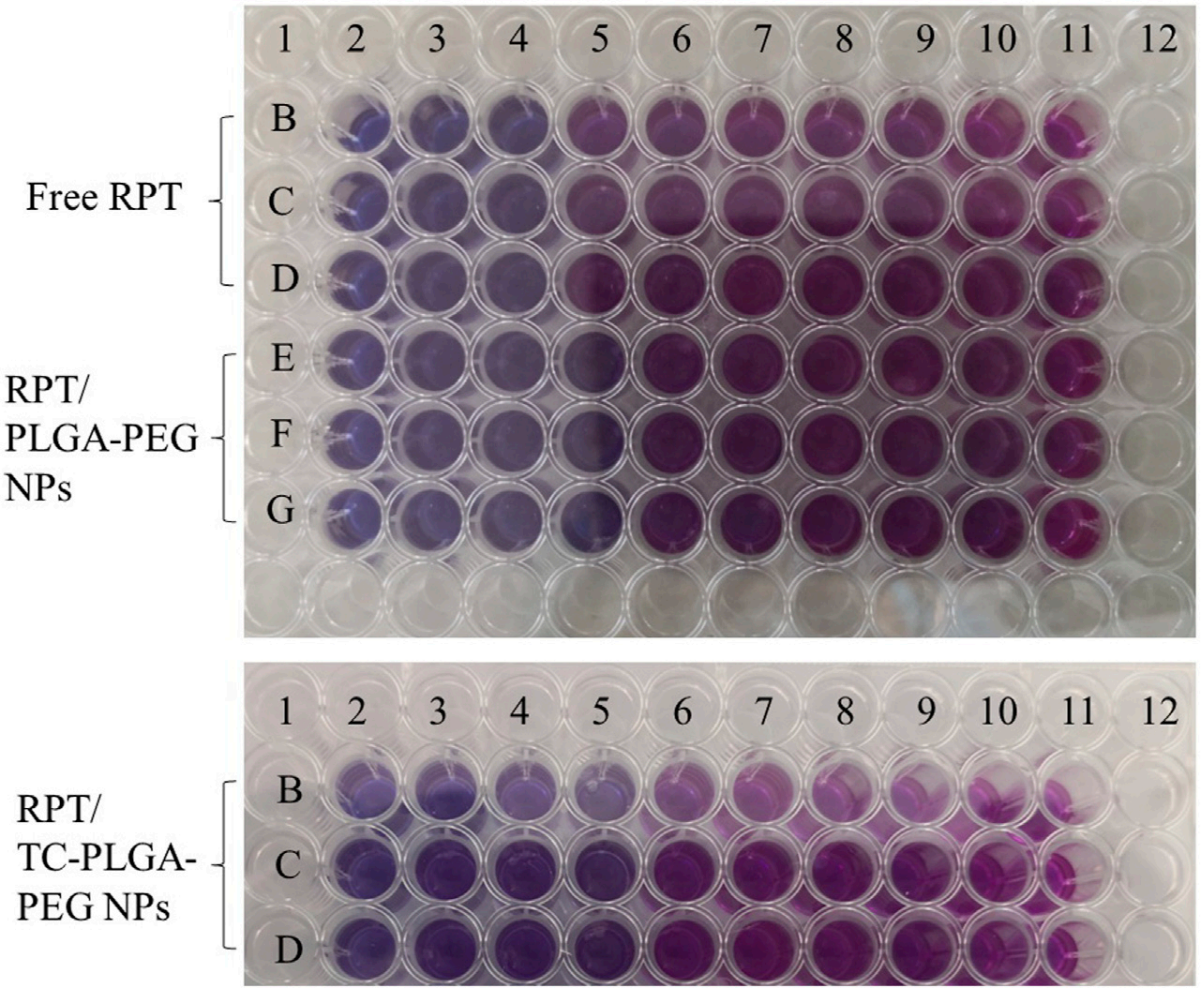
*In vitro* anti-tubercular studies for determination of MIC value.

As shown in Figure 7, the MIC of free PRT was 0.25 μg/mL, whereas this of rifapentine loaded NPs was 0.094 μg/mL (approximately 2.7 lower), indicating that both NPs were more effective than free drug. Furthermore, the MIC of PEG–PLGA NPs was also 0.094 μg/mL, which demonstrated that the conjugated TC has no effects on minimum inhibitory concentration.

### Pharmacokinetics and tissue distribution study

**FIGURE 8:**
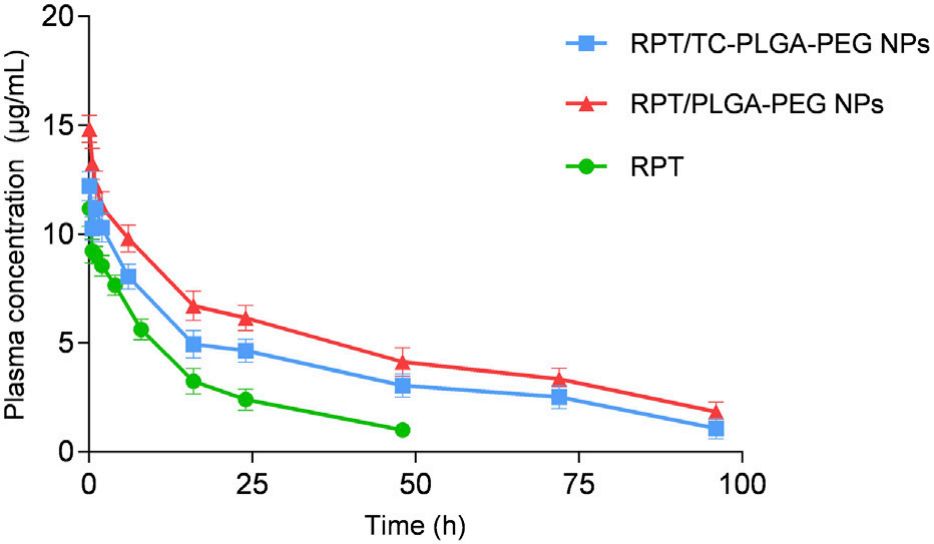
Plasma concentration *versus* time after administration of different drug formulations.

**FIGURE 9:**
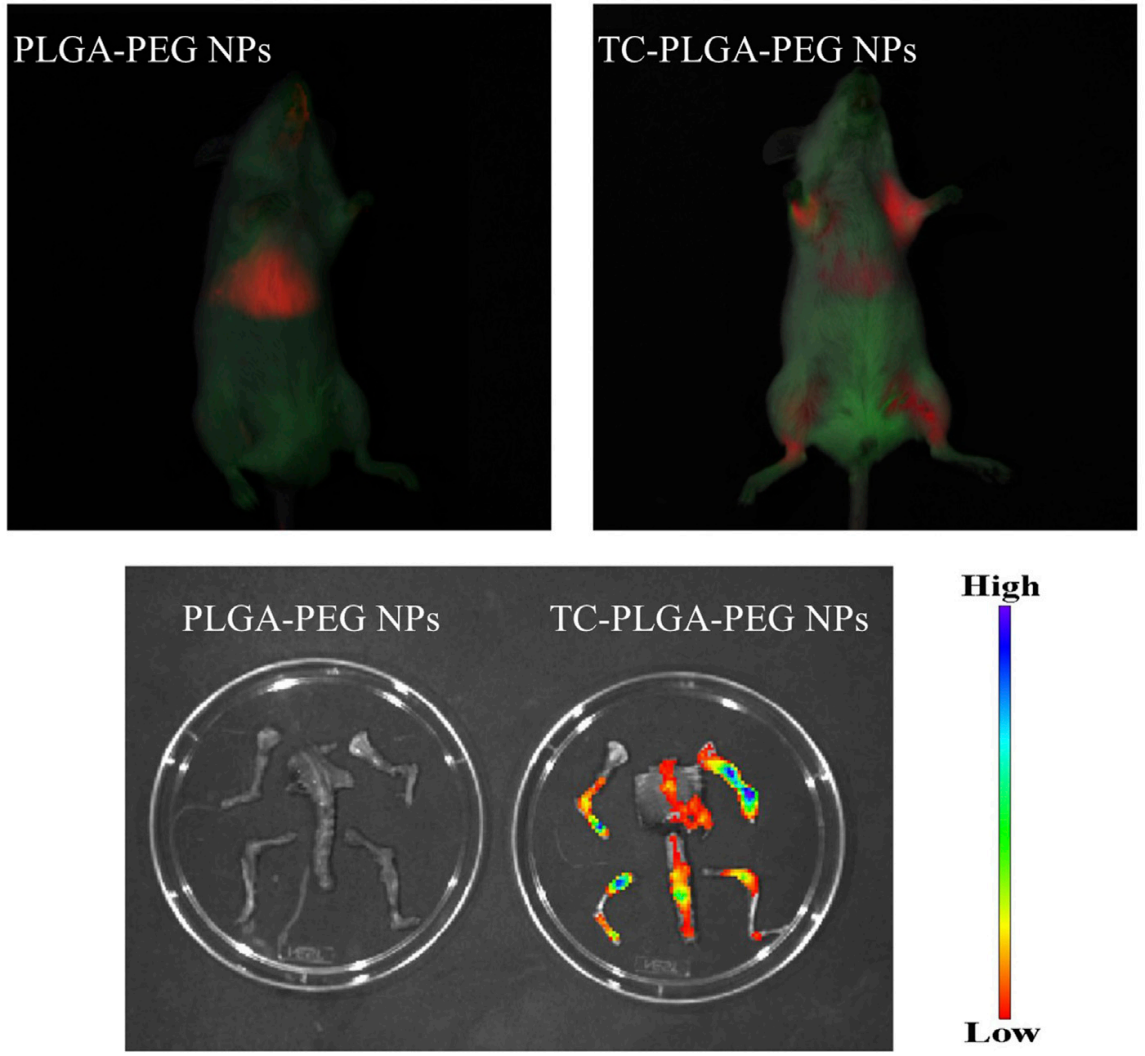
*In vivo* imaging results of fluorescent-loaded NPs.

**FIGURE 10:**
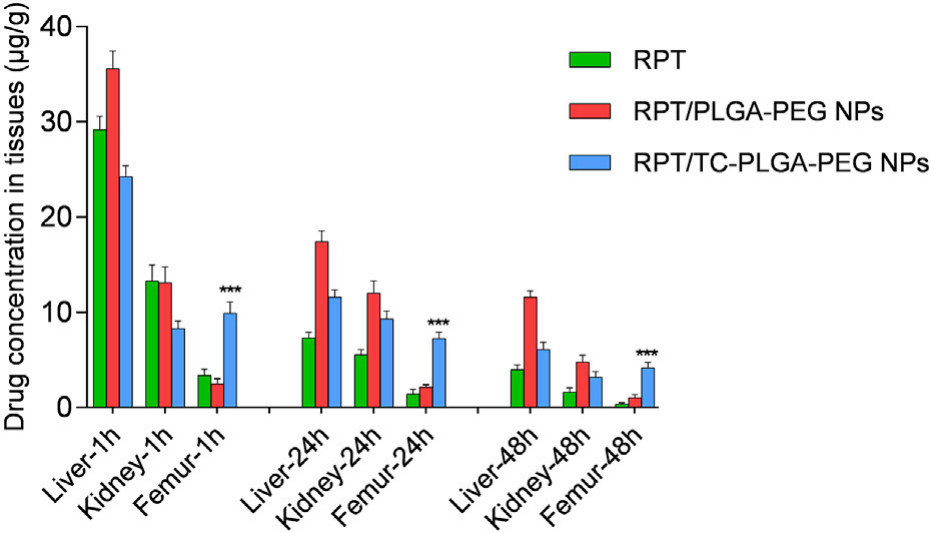
*In vivo* organ distribution in mice.

**FIGURE 11:**
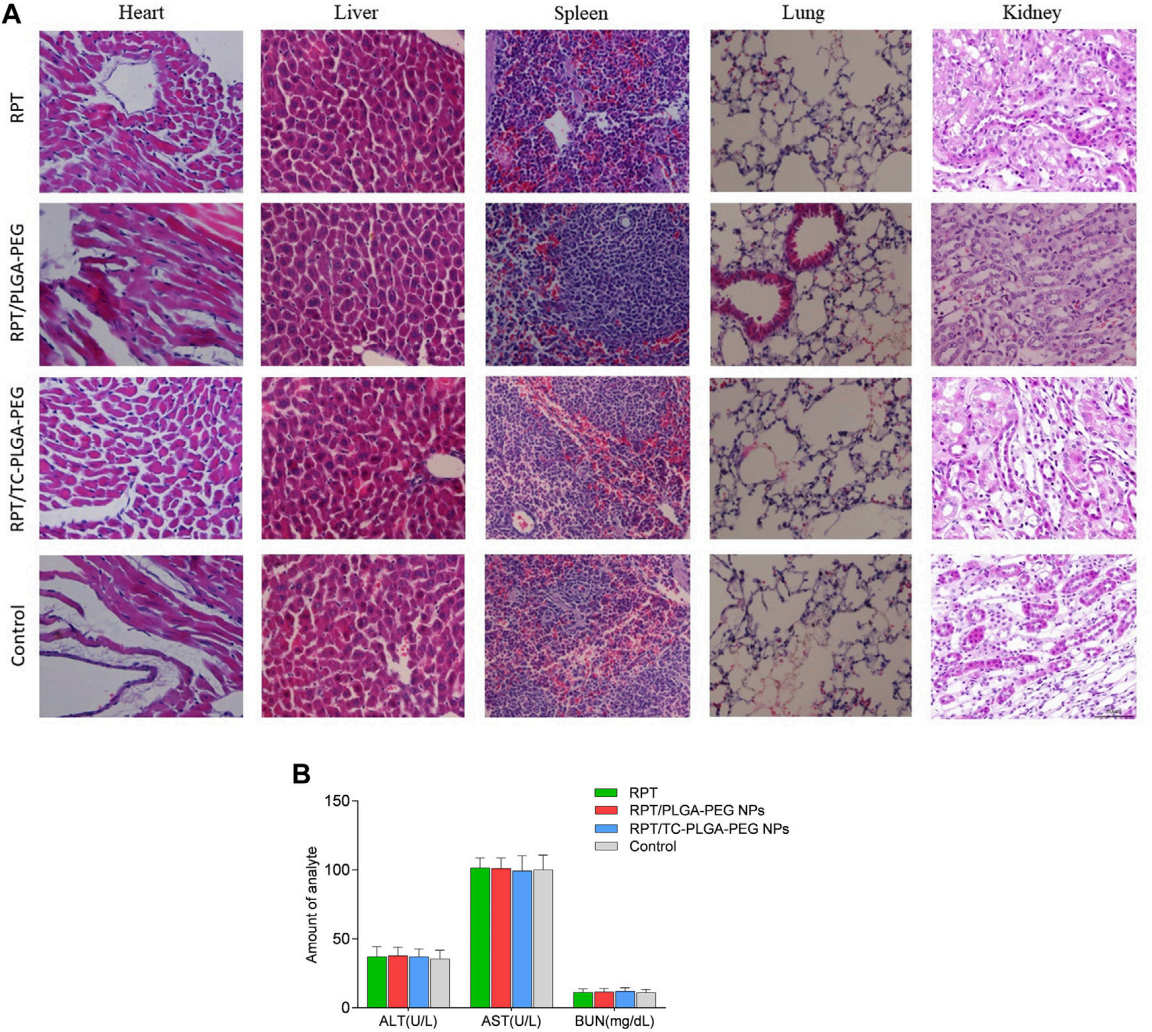
**(A)** H&E staining of the organs of mice. **(B)** Serum biochemistry data on ALT, AST and BUN.

As shown in Figure 8, compared with free rifapentine, both rifapentine loaded NPs showed a prolonged drug release profile for up to nearly 100 h. Table 3 showed the major pharmacokinetic parameters. To further confirm the bone-targeting ability of TC–PEG–PLGA NPs, Dir-loaded NPs were intravenously injected in mice. At 48 h after administration, their bones were collected after *in vivo* imaging acquisition. As shown in Figure 9, bones in the mouse receiving Dir loaded TC-PLGA-PEG NPs exhibited strong fluorescence in comparison to that receiving Dir loaded PLGA-PEG NPs. However, even in the bone-targeting group (TC-PLGA-PEG NPs), NPs were inevitably accumulated in the liver. The results of tissue drug distribution (Figure 10) revealed that the drug concentration of bone tissue in TC–PLGA–PEG group was significantly higher than that in PLGA–PEG group and RPT group at all time (*p* < 0.05). In addition, the drug concentration of TC–PLGA–PEG group in bone tissue was significantly higher than that in blood 24 and 48 h after administration (*p* < 0.05), while the drug concentration of PLGA–PEG group in bone tissue was significantly lower than that in blood 24 and 48 h after administration (*p* < 0.05). The drug concentration of TC–PLGA–PEG group in both liver and kidney was significantly lower than that in blood 1 h after administration (*p* < 0.05). Furthermore, as shown in Figure 11A, there were no obvious abnormalities in the hearth, spleen, liver, lung or kidney. Blood biochemistry analysis was also confirmed that both NPs had no obvious organ toxicity compared with the control group (Figure 11B).

**TABLE 3:** Pharmacokinetic parameters in different groups.

	RPT	RPT/PLGA-PEG NPs	RPT/TC-PLGA-PEG NPs
C_max_ (μg/mL)	11.18 ± 0.82	14.84 ± 0.63	12.21 ± 0.66
*T*_max_ (h)	0.0830 ± 0.00	0.0830 ± 0.00	0.0830 ± 0.00
*t*_1/2_ (h)	20.05 ± 5.38	41.44 ± 8.09	32.68 ± 6.7
AUC_0–∞_ (μg/mL h)	190.65 ± 15.29	590.78 ± 83.30	419.15 ± 73.13
MRT (h)	24.61 ± 5.25	57.78 ± 9.99	46.76 ± 8.69

Abbreviations: TC, tetracycline; PLGA, poly-d,l-lactide-co-glycolide; PEG, poly ethylene glycol; NPs, nanoparticles; C_max_, maximum concentration; *T*_max_, time to reach peak plasma concentration; *t*_1/2_, half life; AUC_0–∞_, area under the curve from 0 to ∞; MRT, mean residence time.

## Discussion

Currently, there have been several reports on various types of nano-delivery systems loaded with anti-tuberculosis drugs. However, only a limited number of these systems have been specifically developed for the treatment of bone tuberculosis. Furthermore, none of these systems possess active bone targeting functionality (Table 4). Ma R et al. reported on a peripheral drug delivery system of bovine serum albumin NPs that can achieve high drug concentration in the affected vertebral body. The concentration of the drug was found to be significantly higher compared to conventional formulations (Ma et al., 2022). This result may be related to the passive targeting of NPs, which differs from the active bone targeting approach employed in the present study.

**TABLE 4:** The summary of nanocarriers designed for bone tuberculosis.

References NO.	Author/Year	Types of delivery system	Methods	Particle size (nm)	Drug-loaded	Encapsulation efficiency (%)	Drug loading (%)	Release time	Designed route of administration
Ge et al. (2018)	Ge et al., 2018	bovine serum albumin NPs	Self-emulsion solvent diffusion	60.5	IHN/RIF	87.8–98	19.8–20.1	5–6 days	Vein injection
Yahia et al. (2023a)	Yahia et al., 2023	bioimplant scaffold	gelatin-based hydrogel incorporating NPs	150/158	LVX/RIF	91.8/49.8	34.3/22.5	30 days/32 days	vertebral implant
Yahia et al. (2023b)	Yahia et al., 2023	3D-printed scaffolds	gelatine-based hydrogel containing silica NPs	84	RIF/LVX	84%	7.5%	60 days	3D-printed vertebral scaffold
Chen et al. (2019)	Chen et al., 2019	chitosan/carbon nanotubes NPs	cross-linking	150–250	INH	-	-	48 h	Vein injection
Prabhu et al. (2021)	Prabhu et al., 2021	mannosylated chitosan NPs	situ gel	142	RIF	73.9	-	12 h	Intra-articular injection
Zhu et al. (2011)	Zhu et al., 2011	mesoporous silica NPs-coated scaffold	gene transfection system	400	IHN/RIF	-	3.8/0.4	30 days	implantable composite scaffold
Liu et al. (2019)	Liu et al	Liposomes-in-hydrogel hybrid system	thin-film dispersion method	130	IHN	89.5	1.3	72 h	Intra-articular injection

Abbreviations: NPs, nanoparticles; INH, isoniazid; RIF, rifampicin; LVX, levofloxacin.

High affinity to bone tissue is the first requirement for a bone-targeted delivery system. The affinity was quantitatively analyzed using HAp powder as model bone, because it is well-documented that HAp is the major constituent of bone tissue. In addition, since bone disease results in the exposure of HAp to the blood, which can be regarded as an ideal target for drug delivery. At present, most of the research studies focus on bone targeting therapy for tumor treatment or managing osteoporosis disease, (Bai et al., 2019), none of these systems possess active bone targeting functionality for the treatment of bone tuberculosis. Therefore, the result reported in the present study confirm the favorable bone-targeted affinity of rifapentine/TC–PLGA–PEG NPs to bone tissue, which is beneficial to increase the amount of rifapentine in bone tissue.

Currently, several studies have shown that TC can be taken up by bone through a reaction that appears to occur directly between TC and HAp. TC was chosen as the targeting moiety to target bone tissue. TC-PLGA-PEG conjugates were synthesized by carbodiimide chemistry between carboxyl group of PLGA–PEG and amino group of TC in the presence of DCC and NHS. The carboxyl group of PLGA was activated with NHS in the presence of DCC. After the addition of TC, NHS was replaced by TC to form the TC–PLGA–PEG conjugates. In the ^*1*^ *H* NMR spectrum of TC-PEG-PLGA, the spectral changes observed after modifying the PLGA-PEG-COOH with TC were primarily characterized by the appearance of peaks at 7.0–8.0 ppm. These peaks can be attributed to the hydrogens of the benzene ring present in TC. In contrast, these peaks were not observed in the ^*1*^ *H* NMR spectrum of PLGA-PEG alone. In addition, the chemical shift range of amide hydrogen is usually between 7.5 and 9.5 ppm, which may partially overlap with the chemical shift range of TC. This can make it challenging to distinguish the chemical shift from the amide bonds of TC-PLGA-PEG. Interestingly, infrared spectrum may provide a better advantage in observing amide bonds, complementing the information obtained from ^*1*^ *H* NMR spectrum. Therefore, based on ^*1*^ *H* NMR, UV and infrared spectrum, it was evidence that the TC-PEG-PLGA conjugates were synthesized successfully.

Que et al. (2022) also created a nano drug delivery system based on TC (TC-mPEG-PLGA micelles) for osteoporotic improvement, which could target bone *in vitro* and *in vivo*. Although the authors confirmed that the micelles group could significantly increase plasma drug concentration, the bone targeting effect of TC could not be well demonstrated because there were only two groups (the free drug group and the TC-mPEG-PLGA micelles group). Similarly, bone-targeted micelles based on TC were successfully fabricated by Xie et al. (2017) and confirmed their good bone affinity *in vitro*, however, it is regrettable that the authors did not further quantify their accumulation *in vivo* through tissue concentration analysis. In addition, compared with −5.7 mv in their study, the higher negative ZP of NPs reported in the present study was considered to be more suitable for increasing stability in the systemic circulation. Some researchers also prepared TC-PLGA NPs for the treatment of osteoporosis (Wang et al., 2015; Zhao et al., 2023), however, it should be noted that the reported average particle size of these NPs was approximately 220 nm, which may not conducive to bone targeting. In contrast, the particle size reported in the present study is suitable for reaching the bone microenvironment through systemic circulation, and its bone targeting ability has been confirmed and quantified both *in vitro* and *in vivo*.

Ideally, it would exhibit a significant difference in particle size due to the modification of PLGA-PEG with TC. However, if there is no significant difference observed, it suggests that certain factors may have influenced the outcome. Potential reasons for this lack of difference include: 1) Both NPs may possess similar physicochemical properties that contribute to their overall stability and size. These properties, such as the hydrophobicity of PLGA and PEGylation, could overshadow the influence of the TC on particle size; 2) The specific modifications made to the nanodrug delivery system, such as surface functionalization with targeting ligand, may not have a substantial impact on particle size. TC modification might primarily affect other aspects, such as targeting efficiency or cellular uptake, rather than particle size; 3) The interactions between TC, PLGA, and PEG could have minimized any potential effects on particle size. The presence of PLGA and PEG molecules may have masked or counteracted the influence of TC on particle size changes; 4) The concentration of the TC could have been suboptimal, resulting in minimal impact on the overall particle size. Therefore, to gain a better understanding of why there was no significant difference in particle size, further investigation and exploration of these factors would be necessary.

At present, polylactic acid and PLGA have been combined with anti-tuberculosis drug to prepare micro- or nano-sized particles for the treatment of bone defects and related infections (Wu et al., 2015; Liang et al., 2020; Wang Z. et al., 2021). Furthermore, it has been demonstrated that these delivery systems can enhance drug concentration at the target site and increase absorption (Ma et al., 2022). Furthermore, PEG was selected as the hydrophilic surface to prolong NPs circulation. In addition to PLGA and PEG, PVA was also applied to prepare NPs. PVA was approved by the FDA and applied for commercial implantation (Chong et al., 2013). More importantly, PVA can be used as an “intermediate” material between hydroxyapatite and rifapentine. Due to hydrogen bonds, PVA can form a film and steadily adhere to hydroxyapatite surface (Chen et al., 2019), which is more conducive to bone targeting. Usually, PVA acts as an efficient steric stabilizer that can reduce interfacial tension and increase emulsion stability, which may keep drug molecules on the interface against its diffusion and accordingly may yield higher EE% (Katas et al., 2009; Saadati and Dadashzadeh, 2014; Maksimenko et al., 2019). reported that the particle size was found to be greatly reduced with increasing PVA concentration from 0.5% to 5%. In the present study, with regard to applying single emulsifier, the NPs prepared by solvent evaporation showed noticeably sustained release behavior when 1% PVA were used, and the concentration of PVA produced NPs with high EE% (>80%).

In the present study, both Rifapentine loaded NPs were prepared through solvent evaporation method, including probe-type ultrasonication and premix membrane emulsification. The premix membrane emulsification is a membrane emulsification method that allows the preparation of monodisperse emulsions with productivities of several orders of magnitude higher than the direct membrane emulsification (Nazir and Vladisavljević, 2021). Compared with our previous study (Liang et al., 2020), the remarkable feature is the preparation of a smaller particle size with bone targeting function. At present, several types of nanocarriers loaded with anti-tuberculosis drugs have been reported. However, studies on rifapentine nano-formulation are few. Recently, Magalhães et al. reported RPT-loaded lipid NPs with drug loading of 2.9% (Magalhães et al., 2020). In contrast, the high %EE in our study demonstrated that the method we used to prepare these NPs was properly selected to favour rifapentine solubility, which may avoid its expulsion during preparation. The particle size plays an important role in drug delivery systems, and it is known that the vasculature in bone have pores of approximately 80–100 nm (Wang et al., 2003; Rafiei and Haddadi, 2017). Therefore, the particle sizes should be less than at least 80 nm to extravasate and be localized in bone after intravenous administration. In addition, the surface charge also affects the fate of NPs in systemic circulation. Since serum proteins are negatively charged, aggregation can occur when positively charged formulations are administered, eventually leading to embolism in blood capillaries. In addition, positively charged NPs have a higher rate of plasma clearance compared with negatively charged NPs (Wang et al., 2010; Yoo et al., 2010). In the present study, the zeta potential of NPs was negative due to the carboxyl end groups of PLGA–PEG chains.

In terms of safety evaluation, excessive haemolysis activity could be seriously life-threatening, thus, haemolytic activity is an important factor to investigate the quality of nano-formulation for intravenously administration. Both NPs were found to possess negligible haemolysis of erythrocytes even at the highest concentrations. Therefore, rifapentine loaded NPs were potentially feasible for intravenously administration. In addition, rifapentine loaded NPs improved the antimycobacterial activity of the rifapentine probably due to a higher contact surface and enhanced penetration.

The prolonged drug release profile is attributed to the role of NPs as long circulating sustained-release drug delivery vehicles. The initial rifapentine release from the matrix occurs by diffusion of the rifapentine from the polymer matrix. There was no significant difference at pre-determined intervals, which demonstrated that the conjugated TC has no effects on rifapentine release. Once rifapentine concentration can be maintained over a longer period of time, this sustained release may be an idea property in the treatment of osteoarticular tuberculosis. Free rifapentine plasma concentration reduced quickly due to rapid distribution and metabolism, leading to short t_1/2_, little rifapentine was detected in the plasma at 24 h after intravenously administration. By contrast, appreciable rifapentine could still be detected in mouse treated with rifapentine loaded NPs at 96 h after administration, leading to the prolonged t_1/2_. Compared with free RPT, the AUC_0-∞_ in both RPT-loaded NPs groups was significantly enhanced, suggesting that NPs could prolong circulation time of RPT through reducing its rapid elimination. Since the PEG molecules that coat the surfaces of PLGA NPs is hydrophilic, it is possible it could prolong NPs circulation. In contrast, the AUC_0-∞_ in RPT/PLGA–PEG NPs group was higher than that in RPT/TC-PLGA-PEG NPs, indicating that more RPT in RPT/TC-PLGA-PEG group may resides in bone tissue. Interestingly, the less t_1/2_ for RPT/TC-PLGA-PEG group compared to RPT/PLGA-PEG group may be attributed to the following reasons: 1) At a constant total drug concentration, a larger proportion of drugs rapidly target bone tissue, resulting in a reduced remaining in the plasma. This phenomenon may also contribute to the lower AUC in RPT/TC-PLGA-PEG group. 2) While the presence of PEG on the surface of PLGA NPs aids in evading metabolism and prolonging the half-life, the modification of NPs with TC might partially counteract the functionality of PEG; 3) It could have been also a cross reaction between TC and RPT, which might reduce the t_1/2_. Therefore, differential Scanning Calorimetry (DSC) should be performed in the future to confirm the chemical nature within the nanocarrier.

To further confirm the bone-targeting ability of TC–PEG–PLGA NPs, Dir-loaded NPs were intravenously injected in mice. Even in the bone-targeting group, NPs were inevitably accumulated in the liver, as typically observed in other studies (Ryu et al., 2016). These results were also in accordance with HAp binding assay that TC modified NPs could bond to the HAp powder at higher density than PLGA-PEG NPs. Interesting, the drug concentration of TC–PLGA–PEG group in both liver and kidney was significantly lower than that in blood 1 h after administration, indicating that it is helpful to reduce the drug toxicity of non-bone tissue organs. The toxicity of organs has always been a limitation in the clinical use of anti-tuberculosis drugs. Although much NPs were taken up and eliminated by ERS organs such as the liver and kidneys (Ganipineni et al., 2018), there were no obvious abnormalities in the hearth, spleen, liver, lung or kidney.

Clinically, since the poor response to existing anti-tuberculosis drugs and low drug concentration in local bone tissues, the traditional drug therapy does not result in satisfactory treatment of osteoarticular tuberculosis. Patients have to take medicine in large doses and high frequency, which leads to early withdrawal of treatment due to severe side effects, eventually result in the emergency of multidrug-resistant tuberculosis. In the present study, rifapentine loaded TC–PLGA–PEG NPs could increase the amount of rifapentine in bone tissue and reduce the amount in other extra-osseous organs, prolong drug release in systemic circulation, enhance anti-tuberculosis activity, and thereby allowing less dosage and frequency for osteoarticular tuberculosis.

## Conclusion

The obtained rifapentine loaded TC–PLGA–PEG NPs successfully combined the bone-targeting properties of TC with the drug delivery characteristics of long-circulating NPs. These NPs were proved to be 48.8 nm in size with good physicochemical characterization. The release of rifapentine from NPs could be maintained for more than 60 h. Most (68.0%) TC-PLGA-PEG NPs could bind to HAp powder *in vitro,* and *in vivo* evaluations also indicated that bone-targeted NPs could improve pharmacokinetic parameters without evident organ toxicity and were preferentially accumulated in bone tissue. More importantly, these NPs were more effective against *M. tuberculosis*. Therefore, we hope that the bone-targeted drug delivery system might improve the therapeutic effect of rifapentine in local bone tissue while reducing both the dose and frequency of rifapentine. *In vivo* studies are needed to investigate these effects for treating osteoarticular tuberculosis.

## Data Availability

The original contributions presented in the study are included in the article/Supplementary Material, further inquiries can be directed to the corresponding authors.

## Abbreviations

ALT, alanine aminotransferase; AST, aspartate aminotransferase; BUN, blood urea nitrogen; MIC, minimum inhibitory concentration; NPs, nanoparticles; PEG, poly ethylene glycol; PLGA, poly-d,l-lactide-co-glycolide; RPT, rifapentine; TC, tetracycline.

## References

[B1] Bai S. B. Liu D. Z. Cheng Y. Cui H. Liu M. Cui M. (2019). Osteoclasts and tumor cells dual targeting nanoparticle to treat bone metastases of lung cancer. Nanomedicine 21, 102054. 10.1016/j.nano.2019.102054 31310809

[B2] Chen G. Wu Y. Yu D. Li R. Luo W. Ma G. (2019). Isoniazid-loaded chitosan/carbon nanotubes microspheres promote secondary wound healing of bone tuberculosis. J. Biomater. Appl. 33 (7), 989–996. 10.1177/0885328218814988 30509120

[B3] Chong S. F. Smith A. A. Zelikin A. N. (2013). Microstructured, functional PVA hydrogels through bioconjugation with oligopeptides under physiological conditions. Small 9 (6), 942–950. 10.1002/smll.201201774 23208951

[B4] Chu W. Huang Y. Yang C. Liao Y. Zhang X. Yan M. (2017). Calcium phosphate nanoparticles functionalized with alendronate-conjugated polyethylene glycol (PEG) for the treatment of bone metastasis. Int. J. Pharm. 516 (1-2), 352–363. 10.1016/j.ijpharm.2016.11.051 27887884

[B5] Ganipineni L. P. Ucakar B. Joudiou N. Bianco J. Danhier P. Zhao M. (2018). Magnetic targeting of paclitaxel-loaded poly(lactic-co-glycolic acid)-based nanoparticles for the treatment of glioblastoma. Int. J. Nanomedicine 13, 4509–4521. 10.2147/ijn.s165184 30127603 PMC6092128

[B6] Ge Z. Ma R. Xu G. Chen Z. Zhang D. Wang Q. (2018). Development and *in vitro* release of isoniazid and rifampicin-loaded bovine serum albumin nanoparticles. Med. Sci. Monit. 24, 473–478. 10.12659/msm.905581 29364864 PMC5791387

[B7] Katas H. Cevher E. Alpar H. O. (2009). Preparation of polyethyleneimine incorporated poly(D,L-lactide-co-glycolide) nanoparticles by spontaneous emulsion diffusion method for small interfering RNA delivery. Int. J. Pharm. 369 (1-2), 144–154. 10.1016/j.ijpharm.2008.10.012 19010405

[B8] Kim K. T. Lee J. Y. Kim D. D. Yoon I. S. Cho H. J. (2019). Recent progress in the development of poly(lactic-co-glycolic acid)-based nanostructures for cancer imaging and therapy. Pharmaceutics 11 (6), 280. 10.3390/pharmaceutics11060280 31197096 PMC6630460

[B9] Li L. Chou K. Deng J. Shen F. He Z. Gao S. (2016). Two-stage total hip arthroplasty for patients with advanced active tuberculosis of the hip. J. Orthop. Surg. Res. 11 (1), 38. 10.1186/s13018-016-0364-3 27029638 PMC4812611

[B10] Liang Q. Xiang H. Li X. Luo C. Ma X. Zhao W. (2020). Development of rifapentine-loaded PLGA-based nanoparticles: *in vitro* characterisation and *in vivo* study in mice. Int. J. Nanomedicine 15, 7491–7507. 10.2147/ijn.s257758 33116484 PMC7547843

[B11] Liu P. Guo B. Wang S. Ding J. Zhou W. (2019). A thermo-responsive and self-healing liposome-in-hydrogel system as an antitubercular drug carrier for localized bone tuberculosis therapy. Int. J. Pharm. 558, 101–109. 10.1016/j.ijpharm.2018.12.083 30634030

[B12] Luque-Michel E. Imbuluzqueta E. Sebastián V. Blanco-Prieto M. J. (2017). Clinical advances of nanocarrier-based cancer therapy and diagnostics. Expert Opin. Drug Deliv. 14 (1), 75–92. 10.1080/17425247.2016.1205585 27339650

[B13] Ma R. Zhang J. Chen Z. Ma H. Liu X. Liang S. (2022). Treatment of spinal tuberculosis in rabbits using bovine serum albumin nanoparticles loaded with isoniazid and rifampicin. Neurol. Res. 44 (3), 268–274. 10.1080/01616412.2021.1979749 34581255

[B14] Magalhães J. Chaves L. Vieira A C. Santos S G. Pinheiro M. Reis S. (2020). Optimization of rifapentine-loaded lipid nanoparticles using a quality-by-design strategy. Pharmaceutics 12 (1), 75. 10.3390/pharmaceutics12010075 31963468 PMC7022298

[B15] Maksimenko O. Malinovskaya J. Shipulo E. Osipova N. Razzhivina V. Arantseva D. (2019). Doxorubicin-loaded PLGA nanoparticles for the chemotherapy of glioblastoma: towards the pharmaceutical development. Int. J. Pharm. 572, 118733. 10.1016/j.ijpharm.2019.118733 31689481

[B16] Mir M. Ahmed N. Rehman A. U. (2017). Recent applications of PLGA based nanostructures in drug delivery. Colloids Surf. B Biointerfaces 159, 217–231. 10.1016/j.colsurfb.2017.07.038 28797972

[B17] Nazir A. Vladisavljević G. T. (2021). Droplet breakup mechanisms in premix membrane emulsification and related microfluidic channels. Adv. Colloid Interface Sci. 290, 102393. 10.1016/j.cis.2021.102393 33770649

[B18] Prabhu P. Fernandes T. Chaubey P. Kaur P. Narayanan S. Vk R. (2021). Mannose-conjugated chitosan nanoparticles for delivery of Rifampicin to Osteoarticular tuberculosis. Drug Deliv. Transl. Res. 11 (4), 1509–1519. 10.1007/s13346-021-01003-7 34021478

[B19] Que Y. Yang Y. Zafar H. Wang D. (2022). Tetracycline-grafted mPEG-PLGA micelles for bone-targeting and osteoporotic improvement. Front. Pharmacol. 13, 993095. 10.3389/fphar.2022.993095 36188546 PMC9515468

[B20] Rafiei P. Haddadi A. (2017). Docetaxel-loaded PLGA and PLGA-PEG nanoparticles for intravenous application: pharmacokinetics and biodistribution profile. Int. J. Nanomedicine 12, 935–947. 10.2147/ijn.s121881 28184163 PMC5291330

[B21] Ryu T. K. Kang R. H. Jeong K. Y. Jun D. R. Koh J. M. Kim D. (2016). Bone-targeted delivery of nanodiamond-based drug carriers conjugated with alendronate for potential osteoporosis treatment. J. Control Release 232, 152–160. 10.1016/j.jconrel.2016.04.025 27094604

[B22] Saadati R. Dadashzadeh S. (2014). Marked effects of combined TPGS and PVA emulsifiers in the fabrication of etoposide-loaded PLGA-PEG nanoparticles: *in vitro* and *in vivo* evaluation. Int. J. Pharm. 464 (1-2), 135–144. 10.1016/j.ijpharm.2014.01.014 24451238

[B23] Sukhithasri V. Vinod V. Varma S. Biswas R. (2014). *Mycobacterium tuberculosis* treatment modalities and recent insights. Curr. Drug Deliv. 11 (6), 744–752. 10.2174/1567201811666140619121728 24947482

[B24] Toft A. L. Dahl V. N. Sifna A. Ige O. M. Schwoebel V. Souleymane M. B. (2022). Treatment outcomes for multidrug- and rifampicin-resistant tuberculosis in central and west Africa: A systematic review and meta-analysis. Int. J. Infect. Dis. 124 (1), S107–S116. 10.1016/j.ijid.2022.08.015 36007688

[B25] Wang B. Wang Y. Hao D. (2021a). Current study of medicinal chemistry for treating spinal tuberculosis. Curr. Med. Chem. 28 (25), 5201–5212. 10.2174/0929867328666201222125225 33355046

[B26] Wang D. Miller S. Sima M. Kopecková P. Kopecek J. (2003). Synthesis and evaluation of water-soluble polymeric bone-targeted drug delivery systems. Bioconjug Chem. 14 (5), 853–859. 10.1021/bc034090j 13129387

[B27] Wang H. Liu J. Tao S. Chai G. Wang J. Hu F. Q. (2015). Tetracycline-grafted PLGA nanoparticles as bone-targeting drug delivery system. Int. J. Nanomedicine 10, 5671–5685. 10.2147/IJN.S88798 26388691 PMC4571930

[B28] Wang J. Sui M. Fan W. (2010). Nanoparticles for tumor targeted therapies and their pharmacokinetics. Curr. Drug Metab. 11 (2), 129–141. 10.2174/138920010791110827 20359289

[B29] Wang Z. Maimaitiaili A. Wang T. Song X. (2021b). Rifapentine polylactic acid sustained-release microsphere complex for spinal tuberculosis therapy: preparation, *in vitro* and *in vivo* studies. Infect. Drug Resist 14, 1781–1794. 10.2147/idr.s304864 34025123 PMC8132576

[B30] World Health Organization (2022). Global tuberculosis report 2022. Available at: https://www.who.int/publications/i/item/9789240061729 .

[B31] Wu J. Zuo Y. Hu Y. Wang J. Li J. Qiao B. (2015). Development and *in vitro* characterization of drug delivery system of rifapentine for osteoarticular tuberculosis. Drug Des. Devel Ther. 9, 1359–1366. 10.2147/dddt.s78407 PMC4357616 25834394

[B32] Xie Y. Liu C. Huang H. Huang J. Deng A. Zou P. (2018). Bone-targeted delivery of simvastatin-loaded PEG-PLGA micelles conjugated with tetracycline for osteoporosis treatment. Drug Deliv. Transl. Res. 8 (5), 1090–1102. 10.1007/s13346-018-0561-1 30027372

[B33] Xie Y. Tan X. Huang J. Huang H. Zou P. Hu J. (2017). Atorvastatin-loaded micelles with bone-targeted ligand for the treatment of osteoporosis. Drug Deliv. 24 (1), 1067–1076. 10.1080/10717544.2017.1347966 28705021 PMC8241047

[B34] Xu Q. Ensign L. M. Boylan N. J. Schön A. Gong X. Yang J. C. (2015). Impact of surface polyethylene glycol (PEG) density on biodegradable nanoparticle transport in mucus *ex vivo* and distribution *in vivo* . ACS Nano 9 (9), 9217–9227. 10.1021/acsnano.5b03876 26301576 PMC4890729

[B35] Yahia S. Khalil I. A. El-Sherbiny I. M. (2023a). Dual antituberculosis drugs-loaded gelatin hydrogel bioimplant for treating spinal tuberculosis. Int. J. Pharm. 633, 122609. 10.1016/j.ijpharm.2023.122609 36642351

[B36] Yahia S. Khalil I. A. Ghoniem M. G. El-Sherbiny I. M. (2023b). 3D-bioimplants mimicking the structure and function of spine units for the treatment of spinal tuberculosis. RSC Adv. 13 (25), 17340–17353. 10.1039/d3ra02351f 37304785 PMC10251188

[B37] Yoo J. W. Chambers E. Mitragotri S. (2010). Factors that control the circulation time of nanoparticles in blood: challenges, solutions and future prospects. Curr. Pharm. Des. 16 (21), 2298–2307. 10.2174/138161210791920496 20618151

[B38] Zhao Z. Deng Y. Deng Y. Chen Z. Zhou Z. (2023). Synthesis and evaluation of bone targeting PLGA nanoparticles loaded with components of traditional Chinese medicine formulas. Recent Pat. Nanotechnol. 17. 10.2174/1872210517666230324103543 36967467

[B39] Zhu M. Wang H. Liu J. He H. Hua X. He Q. (2011). A mesoporous silica nanoparticulate/β-TCP/BG composite drug delivery system for osteoarticular tuberculosis therapy. Biomaterials 32 (7), 1986–1995. 10.1016/j.biomaterials.2010.11.025 21163519

[B40] Zumla A. Chakaya J. Centis R. D'Ambrosio L. Mwaba P. Bates M. (2015). Tuberculosis treatment and management-an update on treatment regimens, trials, new drugs, and adjunct therapies. Lancet Respir. Med. 3 (3), 220–234. 10.1016/s2213-2600(15)00063-6 25773212

